# Kynurenine Pathway of Tryptophan Metabolism in Migraine and Functional Gastrointestinal Disorders

**DOI:** 10.3390/ijms221810134

**Published:** 2021-09-20

**Authors:** Michal Fila, Jan Chojnacki, Elzbieta Pawlowska, Joanna Szczepanska, Cezary Chojnacki, Janusz Blasiak

**Affiliations:** 1 Department of Developmental Neurology and Epileptology, Polish Mother’s Memorial Hospital Research Institute, 93-338 Lodz, Poland; michal.fila@iczmp.edu.pl; 2 Department of Clinical Nutrition and Gastroenterological Diagnostics, Medical University of Lodz, 90-647 Lodz, Poland; jan.chojnacki@umed.lodz.pl (J.C.); cezary.chojnacki@umed.lodz.pl (C.C.); 3 Department of Orthodontics, Medical University of Lodz, 92-217 Lodz, Poland; elzbieta.pawlowska@umed.lodz.pl; 4 Department of Pediatric Dentistry, Medical University of Lodz, 92-216 Lodz, Poland; joanna.szczepanska@umed.lodz.pl; 5 Department of Molecular Genetics, Faculty of Biology and Environmental Protection, University of Lodz, 90-236 Lodz, Poland

**Keywords:** tryptophan metabolism, migraine, kynurenines, functional gastrointestinal diseases, irritable bowel syndrome, aryl hydrocarbon receptor, toll-like receptors

## Abstract

Migraine, the leading cause of disability in the population aged below 50, is associated with functional gastrointestinal (GI) disorders (FGIDs) such as functional nausea, cyclic vomiting syndrome, and irritable bowel syndrome (IBS). Conversely, changes in intestinal GI transit may cause diarrhea or constipation and are a component of the autonomic symptoms associated with pre- and post-dorsal phases of migraine attack. These mutual relationships provoke a question on a common trigger in migraine and FGIDs. The kynurenine (l-kyn) pathway (KP) is the major route for l-tryptophan (l-Trp) metabolism and transforms l-Trp into several neuroactive compounds. Changes in KP were reported in both migraine and FGIDs. Migraine was largely untreatable, but several drugs approved lately by the FDA, including monoclonal antibodies for calcitonin gene-related peptide (CGRP) and its receptor, create a hope for a breakthrough in migraine treatment. Derivatives of l-kyn were efficient in pain relief with a mechanism including CGRP inhibition. KP products are important ligands to the aryl hydrocarbon receptor (AhR), whose activation is implicated in the pathogenesis of GI and migraine. Toll-like receptors (TLRs) may play a role in migraine and IBS pathogeneses, and KP metabolites detected downstream of TLR activation may be an IBS marker. The TLR4 signaling was observed in initiating and maintaining migraine-like behavior through myeloid differentiation primary response gene 88 (MyD88) in the mouse. The aim of this review is to justify the view that KP modulation may provide common triggers for migraine and FGIDs with the involvement of TLR, AhR, and MyD88 activation.

## 1. Introduction

Functional disorders of the gastrointestinal (GI) tract are frequently associated with neurological diseases, including migraine, and are a serious diagnostic problem due to nonspecific syndromes (reviewed in [1,2]). Migraine is bidirectionally comorbid with several other disorders, including neurological, psychiatric, cardio- and cerebrovascular, GI, metaboloendocrine, and immunological diseases [3]. Each of these comorbid diseases shares some mechanism of pathogenesis with migraine and can contribute to the activation of the trigeminovascular system along with the neuroendocrine hypothalamic system, the causative mechanisms in migraine [4]. This reflects the gut–brain axis, a network of complex interactions between the nervous and GI systems with the involvement of the intestinal microbiota. Therefore, the treatment of migraine should be multipathway to identify and eliminate risk and comorbidity factors. Functional disorders of the GI tract may be especially important in this context as the enteric nervous system, cyclic vomiting syndrome, and abdominal migraine directly point to the association between functional GI disorders and migraine headaches. Changes in intestinal GI transit may cause diarrhea or constipation and are a component of the autonomic symptoms associated with pre- and post-dorsal phases of migraine attack [5].

Tryptophan (l-Trp) is an essential exogenous amino acid metabolized in the human body in three main pathways: serotonin (5-hydroxyptamine, 5-HT), kynurenine (l-kyn), and microbiota-related indole pathway. Although the role of 5-HT in the central nervous system (CNS) is well established, the kynurenine pathway of l-Trp metabolism is the main route of l-Trp transformation as it accounts for about 95% of l-Trp metabolism in normal conditions and is implicated in behavioral and cognitive symptoms of neurological diseases [6,7,8].

Recently, we showed that l-Trp metabolism might be implicated in the pathogenesis of lymphocytic colitis, a functional GI disorder [9]. We also reported that the serotonin pathway of l-Trp metabolism is impaired in patients with small bacterial overgrowth (SIBO) [10], a condition which may contribute to the pathogenesis of functional GI diseases [11]. We also showed that older adults with moderate depression had different l-Trp intake and metabolism as compared with controls [12]. Several other works confirm an important role of l-Trp metabolism in functional GI disorders associated with neurological syndromes (reviewed in [13]). These studies suggest that the intake of dietary and supplementary l-Trp may be beneficial in the prevention and therapy of GI disorders associated with neurological symptoms. The GI microbiota may be an important intermediate between l-Trp, GI disorders, and neurological symptoms (reviewed in [14,15]). 

Some reports indicate that increased intake of dietary l-Trp may prevent migraine occurrence or attenuate its GI-related symptoms, such as nausea and vomiting, and photophobia ([14,16]). We observed a decrease in depressive mood disorders after an increase in dietary intake of l-Trp [12].

Migraine therapy is still challenging, but significant progress has occurred lately in this field. Triptans, serotonin 5-HT1B/1D and 5-HT1F receptor agonists, gepants, calcitonin gene-related peptide (CGRP) and its receptor antagonists; ditans, antagonists of metabotropic glutamate receptor 5, and others are used in migraine treatment (reviewed in [17]). The drugs targeting CGRP and its receptors and ligands are a hope for a breakthrough in migraine therapy (reviewed in [18]). Several other antimigraine drugs, apart from anti-CGRP monoclonal antibodies, are considered, including G-protein coupled receptors, glutamate, ion channels, and neuromodulatory devices (reviewed in [19]). A kynurenic acid analogue was reported to abolish nitroglycerin-induced hyperalgesia resulted in an increased CGRP expression in a rat migraine-like headaches model [20,21]. Therefore, expected breakthrough in migraine prevention and treatment may be related to the kynurenine pathway of l-Trp metabolism. Although new antimigraine drugs are still under research, 5-hydroxytryptamine 1 (5-HT1) receptor agonists remain a main group of drugs in migraine prevention and treatment [22]. However, the exact mechanism of the action of 5-HT1 receptor agonists in migraine is not known, and it has been suggested recently that the metabolites of the kynurenine pathway of l-Trp metabolism may be important for this mechanism [23].

The involvement of the gastrointestinal microbiota in the kynurenine metabolic pathway and the activation of this pathway in neurological and psychiatric diseases have recently been reviewed [24,25,26].

Many papers report an association between migraine and functional GI disorders, and most of them underline the need for further research to identify common areas of physiology, but few works show a specific direction of such studies (reviewed in, e.g., [1,2,5,27,28]). 

In this review, we present some general aspects of the role of the gut–brain axis in neurological diseases and these features of the kynurenine pathway of l-Trp metabolism that may be important in functional GI disorders and migraine. Although migraine often associates with organic GI diseases, our review is limited to functional disorders in the GI tract. Consequently, we focus on the impairments in the GI tract that may share pathophysiology with migraine, and not on conditions in which migraine comorbidity may be a secondary symptom, as in many GI cancers [29]. 

## 2. The Gut–Brain Axis

The movement and functions of the GI tract are normally regulated by the brain, although it shows a substantial degree of autonomy due to intrinsic neural plexuses within the enteric nervous system (ENS) [30]. The ENS is a part of the peripheral nervous system controlling GI reaction independently of the CNS [31]. In particular, stomach and esophagus strongly depend on extrinsic neural inputs, particularly from the parasympathetic and sympathetic pathways, contrary to intestines, which can function without extrinsic inputs [32]. Removal of the innervation of the GI tract results in its disfunctions, manifested as nausea and vomiting, abdominal pain, and diarrhea [33]. The gut microbiota may influence the CNS functions and its impairment may cause neurological diseases, including Alzheimer’s disease, mood and anxiety disorders, multiple sclerosis, Parkinson’s disease, and migraine (reviewed in [34]). Therefore, the mutual interaction between the CNS, ENS, and the GI tract justifies the use of the term “gut–brain axis”, which is not limited to the brain, but is extended to the entire CNS.

**Figure 1:**
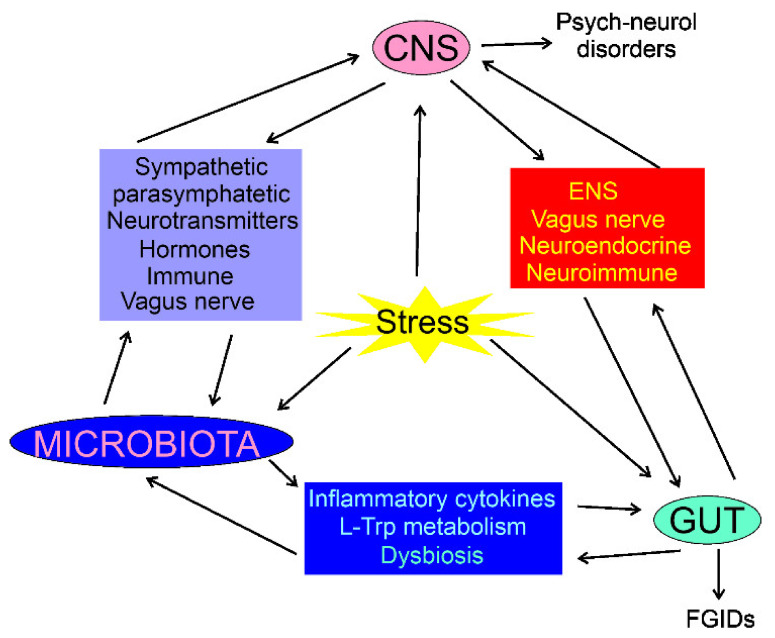
Interaction of the central nervous system (CNS), enteric nervous system (ENS), the gastrointestinal (GI) tract, and the gut microbiota in normal and stress conditions. In each case, the interaction is bidirectional and can be mediated by many factors; some of them are shown. Stress conditions may influence, directly or indirectly, CNS, gut, and microbiota, resulting in psychoneurological disorders and functional GI diseases (FGIDs). The stress may also result in organic GI or CNS diseases, which are not subjected to this review.

The composition of the intestinal microbiota plays a major role in the gut–brain axis as the microbiota-derived neurotransmitters, hormones, and inflammatory mediators may influence CNS functions [35]. The intestinal microbiota may directly modulate the gut–brain axis through the stimulation of the end terminals of the vagus nerve [36]. CNS can modulate the gut microbiota through the sympathetic and parasympathetic systems and by the secretion of neuroendocrine peptides [37]. Physical and psychological stresses may induce alterations in the intestinal microbiota as they promote secretion of the corticotrophin-releasing hormone in hypothalamus, which induces cortisol release from the adrenal glands, resulting in changes in intestine permeability through alterations in the microbiota profile (dysbiosis) (Figure 1) [2]. Therefore, the microbiota can be involved not only in the control of the GI functions, but also in regulating behavior and brain functions (reviewed in [38]). Consequently, the gut–brain axis may be extended to the microbiota–gut–brain axis, but the gut microbiota can be considered as a component of the gut, so the term the gut–brain axis is still justified [39].

The blood–brain barrier (BBB) is a physical and functional separator between the brain and gut/microbiota. Therefore, it is rational to ask how the BBB influences the communication within the gut–brain–microbiota axis. The concept of the BBB has evolved from a static physical barrier between the brain and blood to a part of neovascular unit containing brain microvascular endothelial cells, neurons, microglia, astrocytes, pericytes, and extracellular matrix (reviewed in [40]). Therefore, the BBB can act as an important modulator of the communication within the gut–brain–microbiota axis (reviewed in [41]). Intestinal neuroendocrine cells detect luminal metabolites and send corresponding signals to the brain through the activation of the vagus nerve, release of hormones and neurotransmitters, and through neural routes, including neuroepithelial connections (reviewed in [41]). The mechanisms of the modulation of the gut–brain–microbiota are complex and cannot be thoroughly discussed here due to space limitation. The role of the BBB in the transport of l-kyn and its metabolites is presented further in this review.

As both migraine and functional GI disorders are characterized by pain symptoms, some general information about pain pathogenesis may clarify the interconnection between these two classes of diseases. According to the International Association for the Study of Pain, pain is “an unpleasant sensory and emotional experience associated with, or resembling that associated with, actual or potential tissue damage” [42]. The biogenesis of pain can be underlined by nociceptic, neuropathic, and neuroplastic mechanisms. The kynurenine pathway of l-Trp metabolism may be involved in pain pathogenesis, and the most likely effect associated with this involvement is neuroinflammation (reviewed in [43]). The most common form of pain is nociceptive pain, which is caused by a structural dysfunction, including tissue damage occurring, e.g., in bone fractures (reviewed in [44]). The activation of nociceptors might take place in migraines, and trigeminal perivascular sensory nerve terminals are a prime candidate responsible for this effect (reviewed in [45,46]). Gastrointestinal pain is a form of visceral pain, common in some disorders, including functional GI diseases (reviewed in [47]). In general, GI pain can be nociceptive, neuropathic, and associated with cancer. Mechanisms of GI pain are complex and may include peripheral and central sensitization. Moreover, GI pain may occur with the involvement of the autonomic nervous system, which may be responsible for symptoms associated with GI pain. The exact origin of pain in migraine and functional GI disorders is not completely clear, and is discussed in other recent reviews [48,49].

Nutritional status and diet are reported as critical modifiable factors regulating microbiota both in physiological and pathological conditions (reviewed in [50]). The microbiota is a modifiable target of pharmaceutical intervention, and the gut microbiome, directly and indirectly, may affect drug metabolism [51]. Therefore, the microbiota may play an important intermediatory role between nutritional or pharmaceutical intervention and brain health [39]. In particular, migraine can be considered in therapeutic interventions through targeting the microbiota.

## 3. Metabolism of l-Tryptophan

l-tryptophan is an essential exogenous amino acid that in humans must be provided with diet or as a diet supplement. After uptake, it is included into protein synthesis or undergoes metabolic transformations dependent on the tissue-specific expression of enzymes and cofactors involved in l-Trp metabolism. 

The GI tract is the major site of l-Trp metabolism. There are three main pathways of l-Trp metabolism in humans—the kynurenine pathway, the serotonin pathway, and the microbiota-mediated indole pathway (reviewed in [52]). These pathways are dysregulated in many human pathologies, including neuropsychiatric diseases, and can be targeted in pharmacological interventions (reviewed in [53,54,55]).

**Figure 2:**
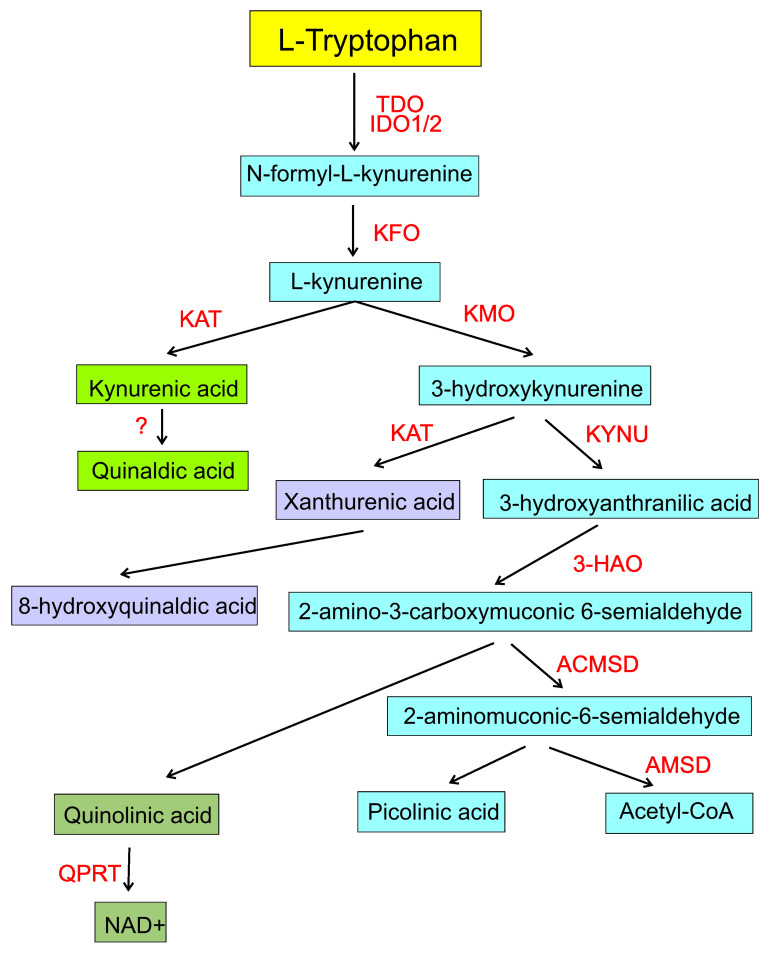
The kynurenine pathway of l-tryptophan metabolism. The key enzymes of the pathway are in red. Different colors in the boxes with metabolites are to distinguish different subpathways only. Some reactions proceed nonenzymatically. NAD+, oxidized nicotinamide adenine dinucleotide; TDO, tryptophan 2,3-dioxygenase IDO1/2, indoleamine 2,3-dioxygenase 1 or 2; KAT, kynurenine aminotransferases; KMO, kynurenine 3-monooxygenase; KYNU, kynureninase; 3-HAO, 3-hydroxyanthranilic acid 3,4-dioxygenase; ACMSD, aminocarboxymuconate-semialdehyde decarboxylase; AMSD, 2-aminomuconic semialdehyde dehydrogenase; QPRT, quinolinic acid phosphoribosyltransferase.

In the kynurenine pathway, occurring mainly in epithelial and immune cells, l-Trp is catabolized to *N*-formyl-l-kynurenine (NFK) by rate-limiting enzymes tryptophan 2,3-dioxygenase (TDO), expressed in the liver, and indoleamine 2,3-dioxygenase 1 and 2 (IDO1/IDO2), expressed in many tissues [56] (Figure 2). NFK is unstable and is immediately metabolized to l-kynurenine (l-kyn) by kynurenine formamidase (KFO), expressed in the liver, kidney, and brain. l-kyn is further metabolized by kynurenine monooxygenase (KMO) to 3-hydroxykynurenine (3-HK), which is subsequently degraded to 3-hydroxyanthranilic acid (3-HAA) by kynureninase (KYNU). Then, 3-HAA is metabolized to 2-amino-3-carboxymuconic 6-semialdehyde (ACMS) by 3-hydroxyanthranilic acid 3,4-dioxygenase (3-HAO). ACMS is cyclized nonenzymatically to quinolinic acid (QUIN) or alternatively converted to 2-aminomuconic-6-semialdehyde (AMS) by 2-amino-3-carboxymuconate-semialdehyde decarboxylase (ACMSD). AMS is metabolized to acetyl-CoA by 2-aminomuconic semialdehyde dehydrogenase (AMSD) or cyclized to picolinic acid (PICA). QUIN can be a substrate for quinolinate phosphoribosyltransferase (QPRT) to produce NAD^+^ (nicotinamide adenine dinucleotide, oxidized form), a cofactor for many enzymes of bioenergetics and genome maintenance [57]. l-kyn may be converted to kynurenic acid (KYNA) in the brain and liver by kynurenine aminotransferase (KAT), which also converts 3-HK to xanthurenic acid (XANA). KYNA may further produce quinaldic acid in an unknown pathway [58].

l-kyn and its metabolites play a role in the regulation of the nervous system and, consequently, in the pathogenesis of neurological and psychiatric disorders (reviewed in [54]). l-kyn is involved in the regulation of the immune reaction as an agonist of the aryl hydrocarbon receptor (AhR), which is a ligand-activated transcription factor with many physiological functions, including regulation of the GI tract and modulation of the gut-brain axis [59,60,61,62,63].

**Figure 3:**
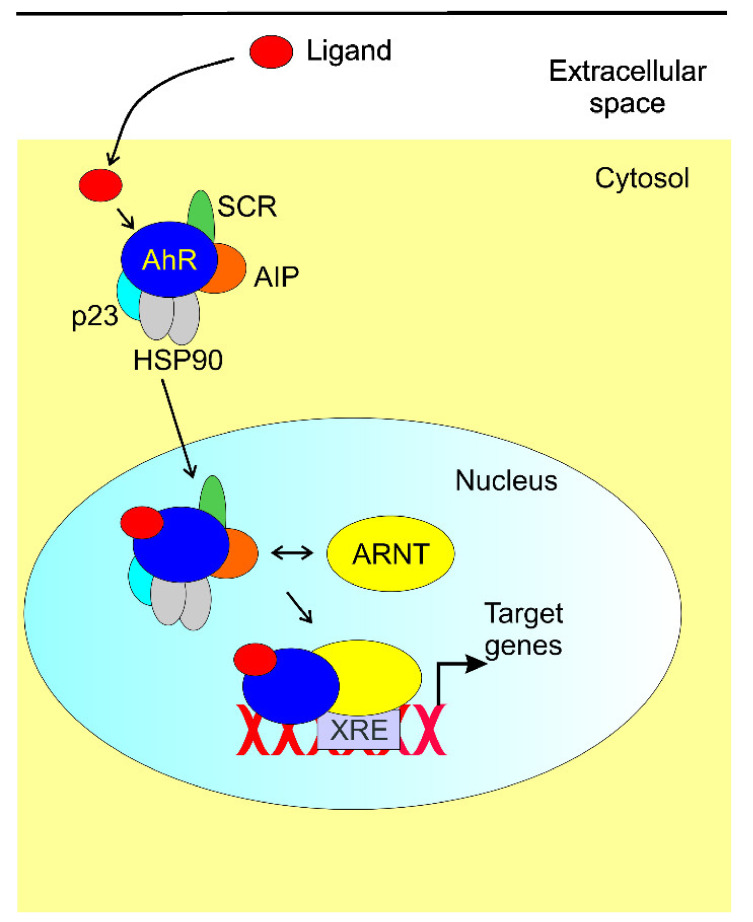
The aryl hydrocarbon receptor (AhR) signaling. Inactive AhR forms a complex with a dimer of 90 kDa heat schock 90. AhR-interacting protein (AIP), the co-chaperone p23, and the protein kinase SRC (SRC proto-oncogene, nonreceptor tyrosine kinase). This complex maintains AhR in an inactive state, prevents its degradation and supports its binding by a ligand. Upon ligand binding, AhR translocates to the nucleus and binds AhR nuclear translocator (ARNT). Then, the complex AhR/ligand/ARNT binds the regulatory region in a myriad of genes having AhR responsive elements (xenobiotic response elements, XREs) to stimulate their transcription led by RNA polymerase II and associated transcription factors (not shown).

In its inactive state, AhR is a part of a protein complex containing a dimer of the 90 kDa heat shock protein (HSP90), AhR-interacting protein (AIP), the co-chaperone p23, and the protein kinase SRC (SRC proto-oncogene, nonreceptor tyrosine kinase) (Figure 3, reviewed in [64]). AhR interaction with the HSP90 dimer maintains its favorable conformation to bind a ligand and prevents its translocation to the nucleus. Ligand binding causes disassociation of the HSP90/AIP/p23 complex from AhR, and its conformation changes to display a stronger affinity to DNA binding and its nuclear translocation. In the nucleus, AhR binds AhR nuclear translocator (ARNT) and regulation of the expression of genes having AhR responsive elements (aka xenobiotic response elements, XREs). AhR may be also involved in the regulation of the genes without XREs [65]. Deficiency in AHR and its ligands impairs the function of intraepithelial lymphocytes (IELs) residing in lamina propria and is important for gut homeostasis, including balanced gut microbiota [66]. Patients with Crohn’s disease were shown to have a suppressed AhR expression in IELs and stromal cells [67]. An anti-inflammatory role of AhR stimulation in dendritic cells was shown [68,69]. These cells show upregulation of IDO1 causing l-kyn production and induction of regulatory T cell production mediated by TGFβ (transforming growth factor β) [70,71,72]. However, subsequent studies with l-Trp metabolites showed that some AhR agonists caused proinflammatory polarization of dendritic cells [73]. These, and other, studies show an important role of AhR in an immunoregulatory gut–brain–microbiome axis mediated by l-Trp metabolism with the involvement of the kynurenine pathway.

KYNA influences brain functions as an antagonist for the 7 nicotinic acetylcholine receptors (7nAchRs) and the *N*-methyl-d-aspartic acid (NMDA) receptor and an agonist for G protein-coupled receptor 35 (GPR35) [74,75,76]. The 3-HK can be neurotoxic through the induction of oxidative stress, and QUIN may be excitotoxic as an NMDA receptor agonist [77,78]. This century has brought a tremendous increase in the interest in the research on KYNA function in the nervous system, with a substantial fraction of studies aiming at manipulation of KYNA production in the brain to prevent and treat disorders of the nervous system.

In the serotonin pathway, l-Trp is a substrate to produce 5-HT, which is about 90% produced in the distal GI tract by enterochromaffin cells and serotonergic neurons of the myenteric plexus that express tryptophan hydroxylase 1 (TPH1) and 2, respectively [79]. TPH1 and TPH2 are rate-limiting enzymes in 5-HT synthesis. The remaining 10% of 5-HT is produced by serotonergic neurons in CNS by TPH2. The 5-HT is inactivated by the serotonin reuptake transporter and degraded to 5-hydroxyindole acetic acid (5-HIAA), which is excreted with urine [80]. The 5-HT is an important intermediator between the GI tract and CNS, involved in intestinal peristalsis, motility, secretion, vasodilatation, and nutrient absorption [32]. The 5-HT is *N*-acetylated by serotonin *N*-acetyl transferase to *N*-acetyl-serotonin, which is then methylated by hydroxyindole O-methyl transferase to melatonin.

Free l-Trp can be absorbed and metabolized by the GI tract microbiota mainly to indole and its acid derivatives, but also to tryptamine, indican, and skatole [81]. Indole and indole acid derivatives are mainly indole-3-aldehyde (IAld), indole-3-acetic acid (IAA), indole-3-propionic acid (IPA), indole-3-acetaldehyde (IAAld), indole-3-lactic acid (ILA), and indole acrylic acid. These microbiota-produced l-Trp metabolites participate in the regulation of the intestinal permeability, inflammation, and host immunity, and some of them are ligands for AhRs. Some dietary molecules directly activate AhR [82]; 5-HT is an AhR antagonist, but 5-HIAA is its agonist [83,84].

## 4. Migraine

Migraine, a common brain disorder with severe headaches and accompanying neurological symptoms, may occur as episodic or chronic disease, with or without aura, which may precede a headache attack, occur during the attack, or occur without a headache [85,86]. Aura happens in about one third of migraine patients and includes visual symptoms, but also prosopagnosia, dyschromatopsia, aphasia, ideational apraxia, alien hand syndrome, and proper name anomia [87]. The migraine prevalence in women is two to three times greater than in men [88].

**Figure 4:**
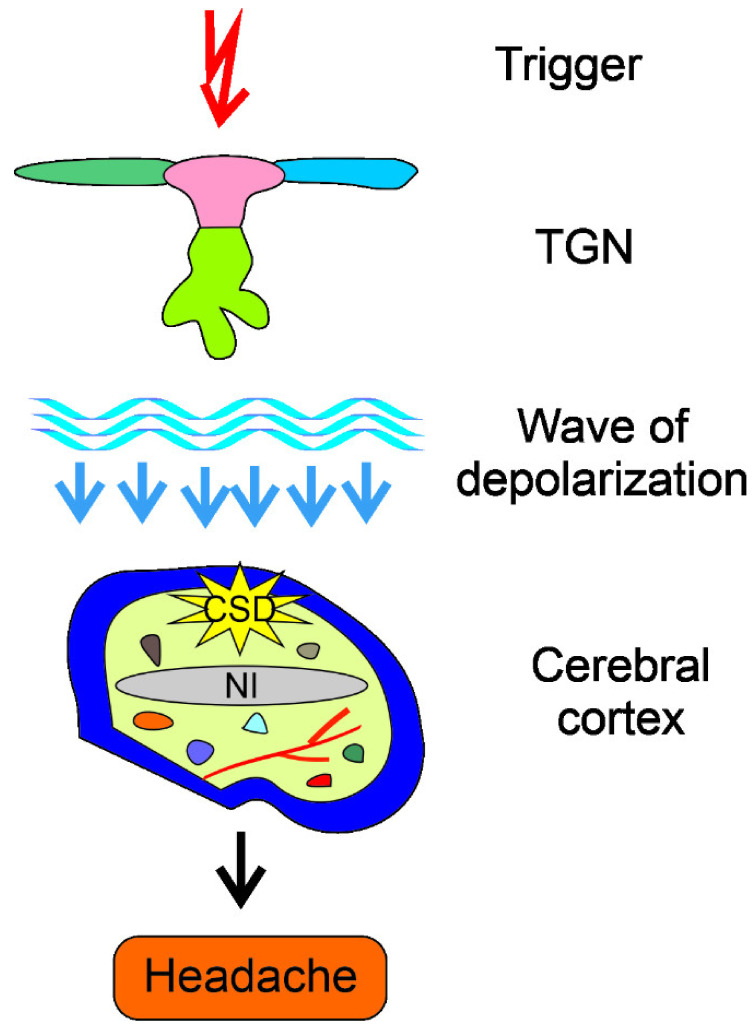
Mechanism of migraine headache induction by a trigger. A migraine trigger (red thunder), which may be of endogenous or exogenous origin, affects the principle sensory nucleus (pink) of the trigeminal nerve with trigeminal ganglion with fragments of the three terminal divisions of the trigeminal nerve (TGN, light green), spinal nucleus (dark green), and mesencephalis nucleus (blue). The trigger-activated trigeminal system sends a wave of depolarization, reaching the cerebral cortex and evoking cortical spreading depression (CSD) and resulting in neurogenic inflammation (NI, symbolized by extended gray oval) and release of inflammatory neurotransmitters (small color objects), which induce dilation of vessels (symbolized by red branched structure), causing the release of pain-producing prostaglandins that induce a migraine headache.

The pathogenesis of migraine is largely unknown and many factors, both genetic/epigenetic and environmental/lifestyle, may be implicated (reviewed in [89]). Migraine is often considered as a threshold disease released by a brain-related trigger (reviewed in [4]) (Figure 4). The trigger and a wave of depolarization induce the activation of the trigeminal nerve (TGN) neurons and the release of chemicals, including calcitonin gene-related peptide (CGRP), substance P, and neurokinin A, inducing dilation of blood vessels and inflammation, directly causing migraine headache attacks. An important element of migraine pathogenesis may be cortical spreading depression (CSD), which is useful in a recurrent episodic or chronic model of migraine [90]. CSD is a slowly propagating wave of depolarization of neurons and glial cells across the cortex, and its association with migraine pathogenesis includes visual aura and the migraine headache [91]. However, some arguments have been raised against the role of CSD in migraine, including certain discrepancies in EEG in migraine and CSD, as well as CSD unknown origin [92].

For a long time, migraine was considered as a vascular disease. That view is justified by the considering alterations in mechanical properties of vessels as the reason for the throbbing, pulsating quality of headache (reviewed in [93]). However, recent decades have provided data showing that changes in vasculatures in the form of vasodilatation are neither necessary nor sufficient to induce migraine attack. Neural imbalance is not only associated with migraine but can be also causative for this disease, and vascular changes are rather a migraine-associated epiphenomenon [94]. Therefore, the neurogenic, rather than vascular, basis of migraine should be considered, but it is rational to include migraine-associated cerebral and meningeal arterial vasodilation into neurogenic effects observed in migraine. However, as mentioned, vasodilation itself might not directly induce migraine, but this does not exclude a role of vessels in migraine pathogenesis. Blood vessels contain cells that release various mediators acting on neurons that may be important in migraine pathogenesis. On the other hand, neurons send signals received by cells in the blood vasculature. Therefore, the vascular and neurogenic bases of migraine unit fit into the neurovascular concept of migraine pathophysiology, and CSD is a mechanism fitting this concept (reviewed in [95]). Prolonged activation of the TGN neurons evoked central sensitization with cutaneous allodynia of the face of migraine patients [96]. The activation of glutamate receptors may be critical for that process, as a correlation between glutamate concentration and face sensory threshold was observed [97].

Gaps in the knowledge of migraine pathogenesis make challenging its prevention and treatment, but some migraine drugs targeting the CGRP signaling pathway, including monoclonal antibodies against the receptor or ligand and small molecule CGRP receptor antagonists, ditans, and gepants approved by the FDA, create a hope for the future [18,62,68]. It is too early to draw a definitive conclusion on newly introduced antimigraine drugs, but a meta-analysis suggests that they may be equally effective as other migraine preventive drugs but have fewer unwanted side effects and are more comfortable to use (reviewed in [98]). However, there are some concerns on the long-term use of these drugs due to the largely unknown role of the target CGRP in other organs [99]. The high cost of these drugs should be also considered in long-term use. Altogether, these concerns provoke a suggestion that these drugs should be applied in refractory migraine patients rather than as first-line treatment [100].

Diet may play an important role in migraine pathogenesis, as many dietary compounds, including chocolate, alcohol, dairy products, fatty and fried foods, tea, coffee, and others, were reported to associate with migraine (reviewed in [101]). Consequently, several kinds of diet, mostly avoidance diets, are considered to prevent migraine attacks [102].

## 5. Kynurenines in the Pathogenesis and Therapy of Migraine

The involvement of the serotonin pathway of l-Trp metabolism in migraine and other neuropsychiatric diseases is reported in much research (reviewed in [103]) and is not subjected to this review. The role of the gut microbiome metabolism of l-Trp in migraine is an emerging issue addressed in several studies (reviewed in [27]).

l-Trp metabolites may act on the glutamatergic system, which is involved in pain transmission, central sensitization, and CSD (reviewed in [104]). The glutamatergic ionotropic and metabotropic receptors are involved in migraine pathogenesis [78,105,106]. A subset of these receptors, NMDA receptors, is important in the onset of CSD and activation of the migraine generator, a brainstem area that is specifically activated during migraine attack [107,108]. Since KYNA was reported to be a competitive antagonist of NMDA receptors whose inhibition protects against glutamate-induced excitotoxicity, it is rational to speculate that the kynurenic pathway of l-Trp metabolism may have a therapeutic potential in migraine [75].

Curto et al. were the first to measure the concentration of the kynurenine pathway products in chronic migraine in humans [109]. They observed a decrease in the levels of l-kyn, KYNA, 3-HK, 3-HAA, 5-HIAA, and QUINA in the serum of migraineurs, as compared to controls. The concentrations of l-Trp, anthranilic acid (ANA), and XANA were significantly higher in these patients. The authors concluded that l-kyn was metabolized in migraine into ANA at the cost of KYNA and 3-HK. As KYNA is a competitive antagonist of NMDA receptors, its reduction supports the hypothesis on the hyperactivity of NMDA receptors in migraine [104]. Increased concentration of XA, which may activate GMR2 (glutamate metabotropic receptor 2), may reflect a balance mechanism to strengthen endogenous analgesic processes. Parallelly, Curto et al. obtained similar results in patients with episodic or chronic cluster headache [110].

Olah et al. studied the effects of KYNA on CSD and BBB permeability in rats [111]. They observed that peripherally administrated KYNA hardly crossed the BBB in control rats, but in the animals with CSD induced by KCl injection, it showed a significantly shorter time at half the amplitude of CSD waves. This important research pointed to the possibility to influence migraine by a systemic administration of KYNA. Chauvel et al. systematically administrated l-kyn to rats to determine whether it could provoke KYNA-induced CSD [112]. In addition, they applied probenecid (PROB) to increase cortical KYNA concentration. They observed that both l-kyn singly and in combination with PROB increased cortical concentration of KYNA in both males and females, and they decreased CSD frequency in females. The latter effect was observed in males only for the l-kyn/PROB combination. The authors concluded that l-kyn intake inhibited CSD, likely by increasing KYNA concentrations in the cortex, and females were more sensitive to that inhibitory effect of l-kyn than males. These results point at a potentially important role of the kynurenine pathway of l-Trp metabolism in migraine and underline the role of sex hormones in migraine prevalence.

Tuka et al. showed decreased plasma concentrations of l-Trp and its metabolites, l-Kyn, ANA, KYNA, 3-HAA, 5 hydroxy-indoleacetic acid, PICA, and melatonin, in the interictal period of episodic migraineurs as compared to controls [113]. This effect was especially strong in patients without aura. The 3-HAA, 5-hydroxy-indoleaceticacid, and melatonin were increased also in the ictal phase of migraine in these patients. A negative correlation was observed between the interictal levels of 3-HAA or melatonin and frequency of attacks. Some other correlations between the concentration of l-Trp metabolism products and the occurrence of attacks were noted. The authors concluded that migraineurs might have a metabolic imbalance manifested by a depressed peripheral l-Trp catabolism during the interictal period, which might act as a trigger of migraine attack.

In conclusion, some associations between migraine occurrence and changes in the kynurenine pathway of l-Trp metabolism were presented. They pointed to general metabolic imbalance in migraine, suggesting the involvement of glutamatergic system and NMDA receptors as mediators between kynurenines and migraine.

## 6. Kynurenines in Irritable Bowel Syndrome

Most studies on the association between the kynurenine pathway of l-Trp metabolism and functional GI diseases are related to irritable bowel syndrome (IBS) (reviewed in [114]). This association seems to be so strong that l-Trp was called an amino acid “essential” in IBS pathogenesis [115]. Clarke et al. observed increases in the l-kyn levels and the l-kyn/l-Trp ratio in male IBS patients as compared with healthy controls [116]. The levels of KYNA and the KYNA/l-kyn ratio decreased in these patients. The authors interpreted their results as the consequence of an increase in the activity of IDO, an immunoresponsive enzyme responsible for l-Trp degradation in the kynurenine pathway in IBS. Ten IBS patients and 26 control subjects were enrolled in that study.

Heitkemper et al. studied sleep quality in female patients with IBS [117]. They observed a lower melatonin/l-Trp level in the IBS patients with predominant diarrhea subgroup as compared with the IBS with predominant constipation and control groups. In addition, they observed that the l-kyn/l-Trp ratios tended to be lower in the total IBS and IBS–diarrhea groups compared to controls. Associations within the control group included melatonin/l-Trp with polysomnography–sleep efficiency and weaker positive correlations with the other ratios for either sleep efficiency or percentage time in rapid eye movement sleep. That study shows that a decrease in melatonin/l-Trp levels may influence sleep quality in IBS patients, especially those with dominant diarrhea.

Keszthelyi et al. showed that IBS patients had significantly lower duodenal mucosal and higher systemic concentrations of both 5-HT and KYNA compared to healthy controls [118]. A positive correlation between mucosal, but not plasma, concentrations of KYNA and 5-HT and psychological state in IBS was also observed. The authors concluded that although both mucosal KYNA and 5-HT decreased in IBS, that did not necessarily mean that an increased activation in the kynurenic pathway resulted in relative 5-HT deficiency. An enhanced release of these substances from the GI tract to the systemic compartment might cause a decrease in intestinal KYNA and 5-HT levels, resulting in disturbance of intestinal homeostasis. Therefore, alterations in a psychological condition of IBS patients might be secondary to changes in GI functions, including kynurenine and/or 5-HT pathway of l-Trp metabolism. An important aspect of that study was the fact that the correlation between l-Trp metabolites and mental conditions in IBS was observed in the GI mucosa and not in the blood.

Altered cytokine production following activation of the toll-like receptor (TLR) family might play a role in IBS pathogenesis (reviewed in [119]). In their next work, Clarke et al. showed that plasma of whole blood from IBS patients cultured with a panel of TLR1-9 agonist displayed a higher l-kyn/l-Trp ratio than controls [116]. The authors demonstrated a distinct downstream profile of l-kyn production after TLR activation in IBS patients compared to controls. That alternated profile included changes in TLR1/2, TLR2, TLR3, TLR5, TLR7, and TLR8. The authors suggested that the kynurenine metabolites of l-Trp detected downstream of TLR activation may be an IBS marker. Interestingly, Ramachandrian et al. observed that TLR4 signaling was important in initiating and maintaining migraine-like behavior and nucleus caudalis neuronal activation through myeloid differentiation primary response gene 88 (MyD88) in the mouse [120]. Earlier it was suggested that the 4 896 A > G polymorphism of the *TLR*-4 gene might be a risk factor for migraine [121]. Therefore, TLR activation may play a role in migraine pathogenesis from the neuroinflammation and the inflammasome perspective, as recently suggested by Kursun et al. [122]. Collins proposed that TLR-4 may be also involved in IBS and psychiatric comorbidity by dysbiosis [123].

Christmas et al. hypothesized that diarrhea-predominant IBS (d-IBS) patients would have elevated levels of l-Trp in plasma because of alterations in l-Trp metabolism [124]. They also checked whether a diet low in l-Trp would reverse this change and reduce symptoms of d-IBS. The d-IBS patients showed higher levels of serum l-Trp and lower tryptophan dioxygenase and total l-Trp oxidation, as measured by the l-kyn/l-Trp to free l-Trp and total kynurenines to free l-Trp ratios. A dairy-free diet neither changed the levels of metabolites of the kynurenine pathway, nor decreased IBS symptoms. The authors concluded that l-Trp metabolism along the kynurenine pathway was inhibited in d-IBS, and a dairy-free diet did not alter that. They hypothesized possible enhanced serotonin activity in d-IBS.

To explore the association between functional GI disorders and neurological symptoms, Farup et al. observed a high prevalence of GI comorbidity in subjects with depression and unexplained neurological symptoms, including headaches [125]. They showed a positive correlation between IBS and the intensity of its symptoms and levels of l-Trp and l-kyn in the cerebrospinal fluid of the subjects. However, the authors’ observations did not confirm earlier studies showing a high l-kyn/l-Trp ratio due to enhanced l-Trp catabolism by the kynurenine pathway in IBS [115,126]. This difference points at an important aspect of studies on neurological diseases associated with functional GI disorders—the target tissue of determination of parameters of interest, including levels of l-Trp and products of its metabolism. We signalized a similar problem describing the work of Keszthelyi et al. [118] and will address this issue in the concluding section.

In summary, the kynurenine pathway of l-Trp metabolism may be involved in IBS pathogenesis by various mechanisms, including TLR stimulation, which may be also important in migraine through MyD88 activation (Table 1).

## 7. Migraine and Functional GI Disorders

According to the Rome IV criteria, cyclic vomiting syndrome (CVS) is a chronic functional disorder of the gut–brain interaction characterized by episodic nausea and vomiting, which should be jointly treated by generalists and specialists (reviewed in [128]). CVS associates with several other functional disorders, such as autonomic dysfunction, anxiety, and depression, but the most pronounced association is with migraine (reviewed in [97,129]).

Aamodt et al. studied associations between headache and GI syndromes in a large questionnaire-based cross-sectional study (the Head-HUNT Study) [130]. A higher prevalence of headache, including migraine, was observed in patients with gastroesophageal reflux disease (GERD), diarrhea, constipation, and nausea as compared with patients without GI disorders. All GI symptoms occurred with the same ratio in patients with migraineous and nonmigraineous headaches, but the association between headache and GI syndromes increased with increased frequency of headache attacks. Similar results for GERD were obtained in two other large studies [131,132].

Nausea and vomiting are considered as syndromes associated with migraine attacks [133]. On the other hand, changes in intestinal GI transit may result in diarrhea or constipation and are a component of the autonomic symptoms associated with pre- and post-dorsal phases of migraine attack [101].

Di Stefano et al. observed a relatively high ratio of migraine occurrence among patients with epigastric pain syndrome (54%) and postprandial distress syndrome (76%) [134]. The authors concluded that migraine may be associated with postprandial hypersensitivity. In a population-based study, Lankarani et al. found a positive association between occurrence of migraine and functional GI disorders, including GERD, IBS, and dyspepsia [135].

Le Gal et al. enrolled children and adolescents diagnosed with either migraine or tension-type headache, as well as age-adjusted children without a history of recurrent headaches [136]. The occurrence of functional GI diseases, diagnosed according to the Rome III criteria, was 32% in the migraine group and 18% in the controls. A significant association between the occurrence of migraine and three GI disorders, functional dyspepsia, IBS, and abdominal migraine, was observed. However, a negative correlation between migraine and functional constipation was noted. The prevalence of functional GI disorders in patients with tension-type headache did not differ from the controls. Therefore, migraine, in contrast to other headache-relate disorders, may be specifically associated with some functional GI diseases, so it is justified to check whether antimigraine treatment may be beneficial for functional GI disorders.

In a similar study, Inaloo et al. observed a higher ratio of headache (Second Edition of The International Headache Classification, ICHD-2) prevalence in children with functional constipation (Rome III criteria) than in the control group—19.8% vs. 5.6% [137]. However, this relationship was significant only in mild, nonmigraine headache subptypes and the authors concluded that it might result from emotional stress, depression, and anxiety, but these results and their interpretation should be considered with as great care as other results on headache in children [138].

Meucci et al. investigated migraine prevalence in patients aged 18–55 years undergoing endoscopic examinations due to dyspeptic symptoms [139]. They observed a higher prevalence of migraine in patients with dysmotility-like dyspepsia as compared with controls, patients with ulcer-like dyspepsia and reflux-like dyspepsia. In addition, a higher ratio of migraine occurrence was observed in patients with nausea and/or vomiting alone as compared with patients of all other groups.

Migraine was associated with *Helicobacter pylori* infection in several studies (reviewed in [1,2]). A meta-analysis including five case–control studies showed a higher *H. pylori* infection in migraineurs than controls (33%) [140]. Stratification on the geographical location indicated a higher prevalence in Asian, but not European, patients, suggesting that other factors beyond the infection can be involved in migraine–*H. pylori* association. Some reports suggest that *H. pylori* eradication may be associated with relief of migraine symptoms [141,142]. However, *H. pylori* infection is an event which is associated with structural changes in the GI epithelial cells and macroscopic changes in the GI epithelium that may progress to GI ulcers and cancers. Therefore, the infection may initiate and progress an organic GI disease, and in this respect, it is rather independent of migraine as any other infection. This statement does not exclude some overlapping areas in pathophysiology of migraine and susceptibility to *H. infection,* which may contribute to observed associations, but a cause–effect relationship between *H. pylori* infection and migraine is, at present, not justified. However, several symptoms associated with *H. pylori* infection, such as immune, inflammatory, and vascular responses, result in the release of immune cells and inflammatory and vasoactive agents into the gastric mucosa which may lower some threshold for migraine and make it an *H. pylori* side effect [109,140]. Finally, when looking for a causal association between *H. pylori* infection and migraine or headaches, several variables that can influence this association should be considered. These include various strains of the bacterium and variation in their distribution in various geographical regions, ethnicity of the population under study, and different subtypes of migraine/headache (reviewed in [28]).

Migraine shares some important features with IBS (reviewed in [143]). Both diseases are chronic, pain-related disorders which occur more prevalent in women than men and associate with psychological comorbidity in terms of fibromyalgia, chronic fatigue syndrome, interstitial cystitis, insomnia, depression, and others. It is estimated that about 60% of migraine patients suffer from allodynia, and most IBS patients report allodynia aside from visceral hypersensitivity [143].

Several studies confirmed the association between migraine and IBS. In a large-scale cohort study, Cole et al. observed 60% higher odds of having migraine, fibromyalgia, and depression in IBS cohort as compared with non-IBS population [114,144]. In another large-scale cohort study in the UK biobank, Chen et al. applied Mendelian randomization analysis with inverse variance weighting and found a weak (OR 1.09, 95% CI 1.01–1.17, *p* = 0.03) causal association between IBS and migraine [145]. In a lesser-scale extent population-based study in a Norwegian cohort, migraine was present about two times more frequently in IBS subpopulation than in controls [146]. Chang et al. summarized six studies on the association between migraine and IBS in a meta-analysis, concluding that 25–50% of patients with IBS had coexisting migraine/headache, whereas only 4–19% of control individuals suffered from this disorder [143]. The odds ratio of having migraine in IBS population was 2.66 in that study. In a later meta-analysis, Wondtrakul et al. considered 11 studies with a total of over 28 thousand migraineurs and over 1.5 million controls [147]. They found that migraineurs had a pooled prevalence of IBS higher by 42% than controls. Many studies report a higher prevalence of IBS among migraineurs than general population [37,135,148,149,150]. Li et al. reported that IBS was more likely in migraine patients with a long headache history, recurrent episodic headache attacks, and anxiety [151]. It was found in a similar study that migraine is a risk factor for future IBS development for patients without comorbid anxiety or depression, but it did not contribute to IBS as an additional risk factor in patients with these comorbidities [152].

In summary, many studies provide convincing evidence on the association between migraine and IBS. A neuroendocrine mechanism is frequently postulated as a common pathophysiological mechanism of migraine and IBS (reviewed in [153]). Cady et al. developed the concept that hostile effects generate a status of hypervigilance in the nervous system, which is linked with an overstated reaction to future threats and episodic attacks of migraine and IBS [154]. They further speculated that this sensitizing reaction might lie in both CNS and ENS, and stated that a connection between migraine and IBS might be uncovered by a disease model of genetically sensitive nervous system changed into hypervigilant one that could develop often disabling and pervasive diseases.

Some studies on the association between migraine and functional GI disorders are summarized in Table 2.

Several works link migraine and functional GI diseases through the gut microbiota. Georgescu et al. observed a strong positive correlation between severity of migraine and dysbiosis assessed in fecal microbiota of IBS patients [156]. The same authors reported that about half of the population of young female migraineurs without aura had gut microbiota dysbiosis [157]. These authors concluded that patients with significant alterations in gut microbiota showed early signs of atherosclerosis and displayed severe migraine disability. Gonzales et al. showed that migraine was positively correlated with higher levels of nitrate-, nitrite-, and nitric oxide-reducing oral microbes in the cohort recruited within the American Gut Project Cohort [158].

Using a wide spectrum of antibiotics and germ-free mice, Tang et al. observed that antibiotics treatment prolonged nitroglycerin (NTG)-induced acute migraine-like pain in wild-type (WT) mice and its blockade by deletion of tumor necrosis factor-alpha (TNFα) or intraspinal trigeminal nucleus caudalis (Sp5C) injection of a TNFα receptor antagonist [159]. Antibiotics treatment prolonged NTG-induced TNFα upregulation in the Sp5C. Probiotics also inhibited the antibiotics-produced migraine-like pain prolongation. NTG-induced migraine-like pain in germ-free mice was markedly enhanced compared to that in WT mice, and gut colonization with fecal microbiota from WT mice strongly inverted microbiota reduction-caused pain increase. In summary, the gut microbiota dysbiosis might contribute to prolongation of migraine-like pain by upregulating TNFα level in the trigeminal nociceptive system. TNFα may induce visceral pain and sensitize afferent endings [160,161].

Several other studies report an association between gut microbiota dysbiosis and migraine, but neither a mechanism underlying this association, nor a causal relationship were reported (reviewed in [162]).

Chen et al. performed a metagenome-wide association study to determine the relationship between gut microbiota and migraine in elderly women suffering from migraine and controls [163]. They observed that the functional module for l-kyn synthesis was enriched in migraineurs. The higher l-kyn degradation module suggested an increased level of its catabolites, including neuroexcitatory QUIN and neuroinhibitory KYNA, both related to CNS diseases [164].

Some results showed that probiotic treatment resulted in a beneficial effect on the frequency and severity of migraine headache attacks (reviewed in [165]). However, more recently, Parohan at al. concluded that pooled analysis of accessible randomized controlled clinical trials suggested that probiotic supplementation had not any significant influence on the frequency and severity of episodic migraine attacks [166].

In summary, migraine can associate with many functional GI disorders, first of all IBS. This association can be mediated by the gut microbiota or occur independently of it.

## 8. Concluding Remarks and Perspectives

Newly developed antimigraine drugs, including anti-CGRP monoclonal antibodies, create a hope for a breakthrough in the treatment of this otherwise untreatable disease. However, it is too early to draw definite conclusions on their efficacy and safety after sufficient long-time observations. The antimigraine action of these drugs can be similar to the effect of a kynurenic acid analogue to abolish nitroglycerin-induced hyperalgesia underlined by an increased CGRP expression. The kynurenine pathway of l-Trp metabolism is reported to be modulated in functional diseases of the GI, and such diseases associate with migraine. Therefore, alteration of the kynurenine pathway may link migraine with functional GI disease, so it may be targeted to efficiently treat both classes of diseases. We do not claim that this the only link between migraine and functional GI diseases. The serotonin pathway of l-Trp metabolism, with melatonin as its final product, is also an established candidate to link these disorders (reviewed in [7]).

The identification of a common trigger in migraine and functional GI diseases is hampered, as both conditions represent many disorders with different pathophysiologies. As many studies on the effects of l-kyn and its metabolites on migraine are performed in laboratory animals, the difference in the kynurenine pathway of l-Trp metabolism in humans and nonhuman species should be explored.

Most of the studies cited in this review were performed in blood of migraine and/or GI diseases patients. This provokes the question of the choice of a proper tissue to investigate the effects of interest. Some discrepancies obtained for GI mucosa and cerebrospinal fluid, as compared with peripheral blood, point out the need for verification of some reported blood-related mechanisms.

Migraine, per se, creates several diagnostic problems that may be enhanced by comorbidities, including functional GI diseases, especially in children and adolescents. Therefore, the interpretation of obtained results in these groups should be performed with great care and double-checked. A differentiation between migraine and nonmigraine headaches is in many cases problematic and, again, comorbidities and young age of patients may affect the right diagnosis. It should be considered that an association between migraine and GI disorders may result from side effects of antimigraine drugs in the GI [131].

Most of the studies on the association between migraine and GI disorders are characterized by a considerable heterogeneity in the data gathering and analysis techniques, which makes a reliable comparison between them hardly possible (reviewed in [153]). Therefore, the data obtained so far do not allow for drawing a definite conclusion on a causative relationship between these two groups of disorders. Functional GI disorders present a wide spectrum of conditions, and the same, although likely in a lesser degree, concerns migraines. Consequently, a general single pathophysiological interpretation of the association between them is questionable. The gut microbiota in both conditions may be an additional causal or interfering factor, and its role in their pathogenesis should be further explored. Therefore, a question could be asked whether it is rational to look for a common “trigger” in these two conditions. Our answer is—yes, it is, as the identification of common areas in the pathophysiology of both classes of diseases would be useful in their diagnosis and therapy.

Most studies on the association between migraine and functional GI diseases and the kynurenine pathway were performed on IBS. Does it mean that this is the main functional GI disease that is associated with migraine? Unfortunately, we could not find too many studies excluding other functional GI diseases from such association. Therefore, there is a need to search for the association between migraine and other functional GI diseases as specified by Rome IV criteria.

The kynurenine pathway of l-Trp metabolism shows a hopeful therapeutic potential in migraine and functional GI diseases, and further experimental studies on animals, as well as clinical studies on individuals suffering from both migraine and functional GI disorders, are needed to determine stages and enzymes of the pathway to be targeted in the therapy of both diseases. It should be considered that the enzymes of the pathway are expressed by specific cell types, and a potential pathological stimulus for their activation/deactivation also shows cell specificity, depending on the affected tissue/organ (reviewed in [53]). Moreover, it should be taken into account that many l-kyn metabolites and enzymes of the kynurenine pathway display two-sided biological properties, depending on their concentration/activity and cellular conditions determined by local environment and tissue type (reviewed in [167]).

Mechanistically, the involvement of kynurenines in migraine and functional GI disorders may be underlined by AhR, TLRs, and MyD888 activation. Due to possible interaction between kynurenines and AhR, AhR signaling can be targeted in the modulation of the kynurenine pathway, but due to the involvement of AhR in cancer transformation, such manipulation should be performed with great care on the target tissue or even cell type [168,169]. Targeting AhR creates some additional problems besides its involvement in carcinogenesis. It is also modulated by the l-Trp metabolites produced in the indole pathway by the gut microbiota.

Although very few studies report an association of changes in the kynurenine pathway in migraine coexisting with functional GI diseases, such changes may provide common triggers for these diseases and may be underlined by the activation of TLR, AhR, and MyD88. Therefore, changes in the kynurenine pathway may belong to the common areas of pathophysiology of both classes of disorders, and so may be a therapeutic target in migraine associated with functional GI disorders.

## Figures and Tables

**Table 1:** Changes in the metabolites of the kynurenine pathway of tryptophan metabolism and associated effects in irritable bowel syndrome (IBS).

Effect	Mechanism	Remarks	Reference
↑l-kyn ^a^, ↓l-kyn/l-Trp, ↓KYNA, ↓KYNA/l-kyn	↑IDO	Male IBS patients, small cohort	[127]
↓melatonin/l-Trp	Unknown	d-IBS compared with c-IBS, lower sleep quality in d-IBS with diarrhea patients	[117]
↓5-HT, ↓KYNA in duodenal mucosa ↑5-HT, ↑KYNA in plasma A positive correlation between mucosal but not plasma concentrations of KYNA and 5-HT and psychological state in IBS	↑release of 5-HT and KYNA from the GI tract to the systemic compartment	Alterations in the psychological condition of IBS patients might be secondary to changes in GI functions	[118]
↑l-kyn/l-Trp	Activation of the TLR family	TLR1/2,2,3,5,7,8 Association of TLR4 with migraine in an animal model	[120,127]
↑l-Trp, ↓l-Trp oxidation	Alteration in l-Trp metabolism, ↓tryptophan dioxygenase	d-IBS patients, dairy-free diet did not change l-Trp or decrease IBS symptoms intensity	[124]
Positive correlation between IBS symptoms and l-Trp and l-kyn patient with depression or unexplained neurological symptoms	Unknown	Determined in cerebrospinal fluid	[125]

^a^—abbreviations: l-kyn, l-kynurenine; l-Trp, l-tryptophan; KYNA, kynurenic acid; IDO, indoleamine 2,3-dioxygenase; 5-HT, 5-hydroxytryptanine, serotonin; TLR, toll-like receptor; d-IBS, IBS with predominant diarrhea; c-IBS, IBS with predominant constipation.

**Table 2:** Association of migraine with functional gastrointestinal (GI) disorders.

GI Disorder	Remarks	Reference
CVS ^a^	Many studies	[129]
GERD, diarrhea, constipation, and nausea	Large-cohort studies	[130,131,132]
Nausea, vomiting	Autonomic symptoms associated with pre- and post-dorsal phases of migraine attack	[155]
Epigastric pain syndrome, postprandial distress syndrome	Associations with postprandial hypersensitivity	[134]
GERD, IBS, dyspepsia	Population-based study	[135]
Positive correlation with functional dyspepsia, IBS and abdominal migraine, negative correlation with functional constipation	Children and adolescent diagnosed with migraine or tension-type headache	[136]
Negative correlation of headaches with functional constipation	Children and adolescent, significant only in nonmigraine subtypes	[138]
Functional dyspepsia	Patients aged 18–55 years, higher migraine prevalence in dysmotility-like dyspepsia as compared with controls, patients with ulcer-like dyspepsia and reflux-like dyspepsia	[139]
IBS	Many studies	[143]

^a^—abbreviations: CVS, cyclic vomiting syndrome; GERD, gastroesophageal reflux disease; IBS, irritable bowel syndrome.
